# Epidemiology of motor vehicle accident-associated ocular trauma

**DOI:** 10.1007/s10792-024-03356-7

**Published:** 2024-11-18

**Authors:** Nishay V. Bhatnagar, Aditya Uppuluri, Neelakshi Bhagat, Paul D. Langer

**Affiliations:** 1 Institute of Ophthalmology and Visual Science, Rutgers New Jersey Medical School, Doctor’s Office Center, Suite- 6100, 90 Bergen Street, Newark, NJ 07103 USA; 2 Rutgers New Jersey Medical School, Newark, NJ USA

**Keywords:** Ocular trauma, Motor vehicle accident, National trauma databank, Orbital fracture, Open globe injury

## Abstract

**Purpose:**

The objective is to investigate trends in cases of motor vehicle accident-associated (MVA-associated) ocular trauma in which the patient was the driver of the motor vehicle.

**Methods:**

The study utilizes data from the 2007–2014 National Trauma Databank (NTDB), a national trauma registry. Status as the driver of the motor vehicle was identified using E-Codes from the International Classification of Diseases, Ninth Revision, Clinical Modification (ICD-9-CM). Trauma diagnoses were identified using D-Codes from the ICD-9-CM. Statistics were performed using IBM SPSS Version 23.

**Results:**

We identified 49,660 cases of ocular trauma secondary to an MVA with a 25.3% increase in injuries over the 8-year time period. Men comprised 68.6% (34,057) of cases. Orbital floor fractures (OFFs) were the most commonly observed ocular injury, occurring in 17,647 (35.5%) cases. There were 2,787 cases of open globe injury (OGI) with the highest proportion of cases in the 65 + age group (6.5%). OGIs were seen in 3.0% of cases with OFFs vs. 7.1% in those without. Drivers under 18 were more likely to have optic pathway/cranial nerve injuries (4.4%) and ocular/adnexal contusions (41.2%) than adult drivers. The mortality rate was 4.3% and was highest in the 65 + age group (9.4%).

**Conclusion:**

Men and young adults comprised the majority of cases of MVA-associated ocular trauma. OFFs were seen in approximately one-third of cases of ocular trauma. OGIs were less commonly observed when a concurrent OFF was observed. Though the overall mortality was 4.3%, there was significant variation by age group.

## Introduction

Motor vehicle accidents (MVAs) are a major cause of death and injury in the United States (US), accounting for 42,939 deaths and an estimated 2,497,657 injuries in 2021 [1]. These fatal injuries are preventable and cause a significant burden in healthcare [1–3]. Deaths related to MVAs have increased 18% since 2019. It is estimated that 1.14% of all MVA-associated emergency department (ED) visits involve ocular trauma [4]. Commonly, MVA -related ocular trauma occurs due to airbag deployment, broken glass/windshield, and/or lack of proper seatbelt usage [5, 6]. Additionally, of all ocular trauma cases, approximately 30% are caused by motor vehicles, making MVA-related injury one of the most common causes of ocular trauma cases [7]. Of these MVA-related cases, most occurred to the driver of the motor vehicle compared to passengers or pedestrians when the driver status was known [7, 8].

Only a few studies have investigated the demographics associated with MVA-associated ocular trauma, and to our knowledge, no large study has analyzed trends of these traumatic ocular injuries over the last 2 decades [4, 7, 8]. This study aims to investigate trends in motor vehicle related ocular injuries sustained by the driver of the vehicle utilizing the National Trauma Databank (NTDB) from 2007 to 2014.

## Methods

The data were obtained using NTDB, a national trauma registry that collects information pertaining to trauma-related hospital admissions and deaths that present to member institutions [9]. Currently, there are over 700 participating hospitals, with 20–30% classified as Level I trauma centers. The NTDB can be accessed at this link: https://www.facs.org/quality-programs/trauma/quality/national-trauma-data-bank/. This study received exemption by the Rutgers University Institutional Review Board due to non-human determination according to the National Bureau of Economic Research.

This analysis used de-identified information from the NTDB from 2007 to 2014. E-Codes from the International Classification of Diseases, Ninth Revision, Clinical Modification (ICD-9-CM) were used to sort and identify the patient as the driver of the motor vehicle (810.0, 811.0, 812.0, 813.0, 814.0, 815.0, 816.0). Non-drivers of motor vehicles were excluded from the study because the NTDB does not have specific qualifiers for passengers in a motor vehicle and could be confounded with pedestrians or cyclists. The minimum age included in this study was 14 years of age since that is the youngest age that one can obtain a driving learner’s permit in some states in the US [10]. Within these inclusion criteria, trauma diagnoses were identified using the D-Codes from the ICD-9-CM to identify subjects with MVA-associated ocular trauma. The included codes are as follows: burn (940.0–940.9), optic pathway/cranial nerve injury (951.0–951.4, 950.0–950.3, 950.9), open globe injury (871.0–871.2, 871.4–871.7, 871.9), orbital floor fracture (802.6, 802.7), open wound ocular adnexa (870.0–870.9), ocular/adnexal contusion (921.0–921.9), superficial eye injury (918.0–918.9), non-orbital skull fracture (800.0–802.5, 802.8–804.99), spine/trunk fracture (805.0–809.99), upper limb fracture (810.0–819.99), lower limb fracture (820.0–929.99), and intracranial hemorrhage (853.0-853.99, 430.0–423.99).

### Statistical analysis

The variables describing the demographics of the driver of the vehicle who sustained an ocular injury were collected and included age, gender, race and ethnicity, type of orbital trauma, blood alcohol test results, and seatbelt usage. Age (in years) was categorized into 14–17, 18–64, and 65 + to evaluate how newer drivers and senior drivers may impact rates of ocular injury. Ocular trauma was classified as: optic pathway/cranial nerve injury, orbital floor fracture, open wound of ocular adnexa, ocular/adnexal contusion, open globe injury, and superficial eye injury.

Statistical analyses were performed using IBM SPSS Version 23. Independent Samples T-Testing and Chi Square Testing were used to compare groups; p-values less than 0.05 were considered statistically significant.

## Results

**Fig. 1:**
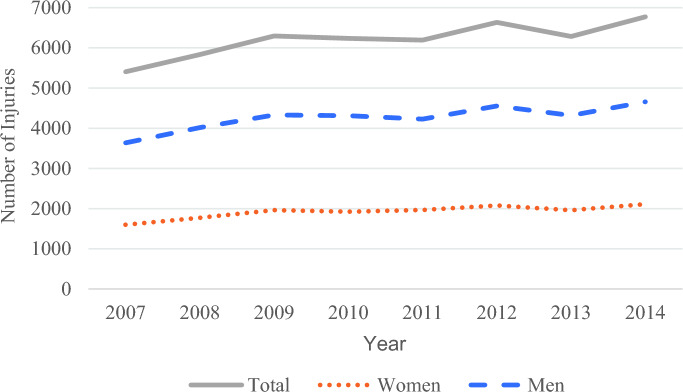
Cases of ocular trauma between 2007 and 2014

**Fig. 2:**
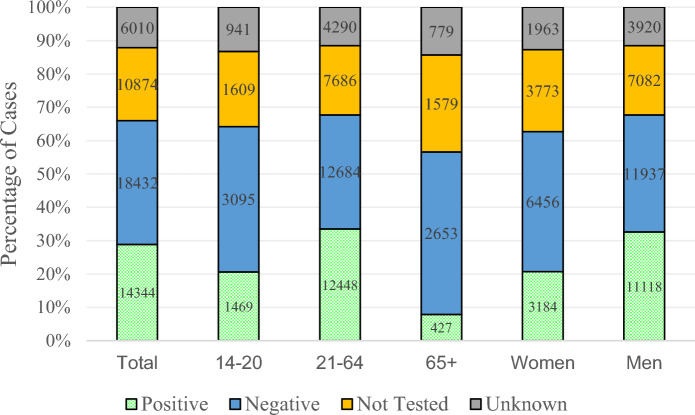
Comparison of blood alcohol content tests between patient demographics

A total of 49,660 cases of MVA-associated ocular trauma were identified between 2007 and 2014 in which the patient was the driver of the motor vehicle. Ocular injuries increased by 25.3% over the 8-year time period, with 2014 having the most ocular trauma cases of 6,774 (Fig. 1) Most ocular injuries (85.0%) occurred in the 18–64 age group, and young drivers (14–17) accounted for 4.1% of cases (Table 1). By gender, the majority of cases (68.6%) comprised men (Table 1). When comparing race and ethnicity, 12.5% of patients identified as Black and 10.5% of patients identified as Hispanic (Table 1). A total of 28.9% of patients were tested and had a positive Blood Alcohol Content (BAC) test, with positive BAC tests more common in people ages 21–64 (33.5%) and men (32.6%) (Fig. 2). Seatbelt usage was confirmed in 45.3% of cases, while 26.7% reported not wearing a seatbelt. By gender, 41.9% of male patients wore seatbelts while 53.4% of female patients wore seatbelts (*p* < 0.001). Additionally, of the patients with ocular trauma, seatbelts were worn in 45.9% of non-fatal cases, contrasted with 31.9% of seatbelt usage in those who died (*p* < 0.001).

**Table 1:** Patient demographics of ocular trauma cases

Demographic	Total Cases (*n* = 49660)	Percentage (%)
Age (yrs.)		
14–17	2024	4.08
18–64	42,198	84.97
65+	5438	10.95
Gender		
Women	15,376	30.96
Men	34,057	68.58
Unreported/unknown	227	0.46
Race		
Asian	769	1.55
Black	6212	12.51
Native American	349	0.7
Native Hawaiian/Pacific Islander	93	0.19
White	35,034	70.55
Other/not reported	7203	14.5
Ethnicity		
Hispanic or Latino	5214	10.5
Not Hispanic or Latino	44,446	89.5

**Fig. 3:**
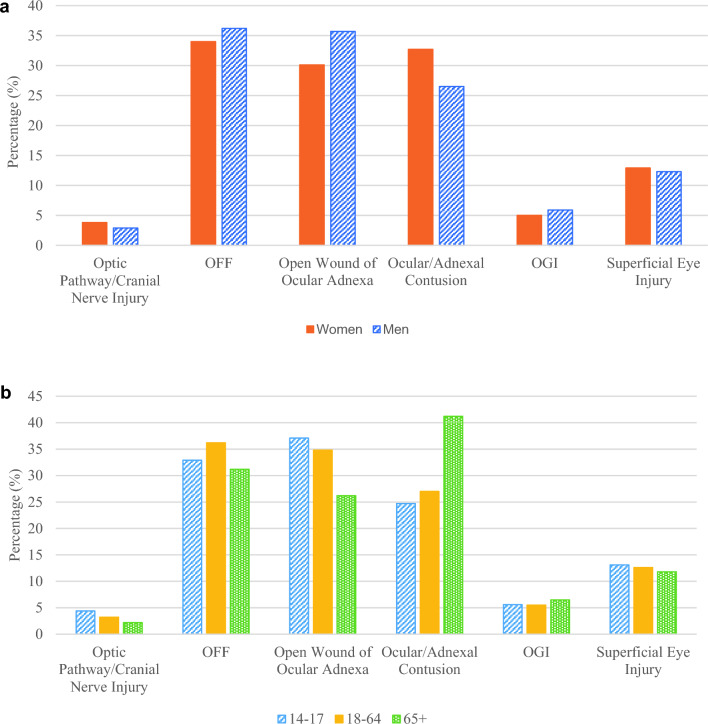
Proportions of ocular trauma cases by gender (**a**) and age (**b**)

In descending order, the most frequent types of ocular trauma observed were orbital floor fractures (OFF) (35.5%), open wound of ocular adnexa (40.0%), ocular/adnexal contusion (28.5%), superficial eye injury (12.5%), open globe injury (OGI) (5.6%), and optic pathway/cranial nerve injury (3.2%) (Table 2). On average, 3.0% of OFF injuries were accompanied by OGIs, while 7.1% of patients without OFFs had an OGI. By gender, open wounds of ocular adnexa, OFFs, and OGIs were found to be more common in men while optic pathway/cranial nerve injuries and ocular adnexal contusion were more common in women (Fig. 3a). Younger patients (14–17 cohort) were more likely to have optic pathway/cranial nerve injuries (4.4%, *p* < 0.001) and open wounds of ocular adnexa (37.1%, *p* < 0.001) while older patients (65+) were more likely to get ocular/adnexal contusions (41.2%, *p* < 0.001) (Fig. 3b) Concurrent non-orbital floor facial bone fractures were observed in 50.6% of patients; spine/trunk fractures in 48.7%, and intracranial hemorrhages in 16.1% of cases.

**Table 2:** Distribution of ocular trauma cases by demographics

Demographic	Ocular trauma					
	Optic pathway/cranial nerve injury	OFF	Open wound of ocular adnexa	Ocular/adnexal contusion	OGI	Superficial eye injury
Total	1576 (100%)	17,647 (100%)	16,868 (100%)	14,130 (100%)	2787 (100%)	6215 (100%)
Age (yrs.)						
14–17	90 (5.7)	665 (3.8)	750 (4.4)	500 (3.5)	114 (4.1)	265 (4.3)
18–64	1368 (86.8)	15,288 (86.6)	14,695 (87.1)	11,389 (80.6)	2318 (83.2)	5309 (85.4)
65+	118 (7.5)	1694 (9.6)	1423 (8.4)	2241 (15.9)	355 (12.7)	641 (10.3)
Gender						
Women	579 (36.7)	5222 (29.6)	4621 (27.4)	5031 (35.6)	762 (27.3)	1986 (32.0)
Men	991 (62.9)	12,339 (69.9)	12,172 (72.2)	9035 (63.9)	2015 (72.3)	4195 (67.5)
Unreported/ unknown	6 (0.4)	86 (0.5)	75 (0.4)	65 (0.5)	10 (0.4)	34 (0.5)

The average length of hospital stay was 8.2 days (median = 4.0 days) and was longest in the 65 + age group (9.1 days) and shortest in the 14–17 age group (6.9 days). Of these patients, 46% required admission to the intensive care unit for additional care, with an average length of stay of 7.4 days (median = 4.0 days). Approximately 26.7% of patients required ventilator use, and the average length of ventilator use was 7.4 days (median = 4.0 days). The overall mortality rate was 4.3%. Women between the ages of 18 and 64 had the lowest mortality rate, while men over the age of 65 had the highest (9.8%) (Table 3).

**Table 3:** Demographics of patient stay in the hospital

Demographic	Average hospital length of stay (days)	Percent admitted to ICU	Average ICU length of stay (days)	Percent ventilator use	Average ventilator use time	Mortality (%)
Total	8.2	46.3	7.4	26.7	7.4	4.3
Age (yrs.)						
14–17	7.0	45.7	6.8	26.1	6.8	3.7
18–64	8.1	45.1	7.3	26.7	7.2	3.6
65+	9.1	55.5	8.0	26.2	9.5	9.4
Gender						
Women	7.8	44.0	6.9	23.7	7.2	4.1
Men	8.3	47.3	7.6	28.0	7.5	4.4
Unreported/ unknown	9.1	46.3	7.8	23.3	8.0	1.8

## Discussion

This study observed the trends and types of ocular trauma sustained by the driver of the vehicle in motor vehicle accidents that presented to the ED from 2007 to 2014. A total of 49,660 cases were observed, with a 25.3% increase in these cases over this time 8-year time period (Fig. 1).

By gender, the MVA-associated ocular injuries were more common in men than women (Table 1). Men suffered more severe ocular trauma injuries (OGIs and OFFs) in MVAs compared to women drivers. They also had a longer mean length of stay in the hospital with a higher mortality rate than women hospitalized with MVA-related ocular injuries (Tables 1 and 3). Increased risk behaviors (driving under the influence, not using seat belts) in men while driving may be related to the increased proportion of MVA-related ocular injury noted in men [11]. This hypothesis is supported by a higher proportion of men having positive BAC tests and lower seatbelt usage than women (Fig. 2). These results are similar to those of previous studies that found an association with MVA-related ocular trauma and the male gender [7, 8].

Open wounds of ocular adnexa, OFFs, and ocular/adnexal contusions were the most common forms of injury, while superficial eye injuries, OGIs, and optic pathway/cranial nerve injury were less common. These patterns are usually observed with blunt trauma injuries [7, 12]. Lower prevalence of OGIs was noted in subjects with OFFs than in absence of OFFs, which is consistent with reports in the literature [13].

The mean duration of hospital stay for MVA-associated ocular injuries in this study was higher than the 5.6 days reported for overall MVA-related injuries in the literature [14]. These ocular trauma cases had a high prevalence of associated non-ocular injuries, with spine/trunk fractures seen in nearly half of the cases. These findings suggest that MVA-associated ocular trauma cases are complicated with systemic injuries.

This study has several limitations. The NTDB contains data from only a fraction of all trauma programs, and therefore it may not be representative of the general population that presents to a trauma site [9]. As such, extrapolations or comparisons to a broader general population may not be valid. Still, over 65% of national Level I trauma centers participate in the registry, lending credence to the data. Also, retrospective studies in general are subject to data collection errors, including potential misclassification of variables, incomplete documentation or errors in ICD-9-CM coding, and the inability to prove any causality between demographics and outcomes.

## Conclusion

This study was designed to analyze the demographics of MVA-associated cases involving ocular trauma since no large studies have analyzed these trends over the past two decades [4, 7, 8]. In summary, MVA-associated ocular trauma cases were shown to increase nationally in the time period from 2007 to 2014, with most cases occurring in people ages 18–64 and in men. By proportion, OFFs and OGIs most commonly occurred in the 18–64 and 65 + age groups, respectively. Additionally, people age 65 + were more likely to have longer hospital stays, and people age 65 + and male had a higher mortality rate. Recognizing these trends in MVA-associated ocular injury can serve to improve management of patients in motor vehicle accidents and better inform public policy initiatives designed to decrease ocular injuries occurring in MVAs.

## Data Availability

No datasets were generated or analysed during the current study.
